# Complete genome sequence of *Shigella flexneri *5b and comparison with *Shigella flexneri *2a

**DOI:** 10.1186/1471-2164-7-173

**Published:** 2006-07-06

**Authors:** Huan Nie, Fan Yang, Xiaobing Zhang, Jian Yang, Lihong Chen, Jing Wang, Zhaohui Xiong, Junping Peng, Lilian Sun, Jie Dong, Ying Xue, Xingye Xu, Shuxia Chen, Zhijian Yao, Yan Shen, Qi Jin

**Affiliations:** 1 College of Biological Sciences China Agricultural University, Beijing 100094, China; 2 State Key Laboratory for Molecular Virology and Genetic Engineering, Beijing 100052, China; 3 National Center of Human Genome Research, Beijing 100176, China; 4 Institute of Pathogen Biology, Chinese Academy of Medical Sciences, Beijing 100730, China

## Abstract

**Background:**

*Shigella *bacteria cause dysentery, which remains a significant threat to public health. *Shigella flexneri *is the most common species in both developing and developed countries. Five *Shigella *genomes have been sequenced, revealing dynamic and diverse features. To investigate the intra-species diversity of *S. flexneri *genomes further, we have sequenced the complete genome of *S. flexneri *5b strain 8401 (abbreviated Sf8401) and compared it with *S. flexneri *2a (Sf301).

**Results:**

The Sf8401 chromosome is 4.5-Mb in size, a little smaller than that of Sf301, mainly because the former lacks the SHI-1 pathogenicity island (PAI). Compared with Sf301, there are 6 inversions and one translocation in Sf8401, which are probably mediated by insertion sequences (IS). There are clear differences in the known PAIs between these two genomes. The bacteriophage SfV segment remaining in SHI-O of Sf8401 is clearly larger than the remnants of bacteriophage SfII in Sf301. SHI-1 is absent from Sf8401 but a specific related protein is found next to the *pheV *locus. SHI-2 is involved in one intra-replichore inversion near the origin of replication, which may change the expression of *iut/iuc *genes. Moreover, genes related to the glycine-betaine biosynthesis pathway are present only in Sf8401 among the known *Shigella *genomes.

**Conclusion:**

Our data show that the two *S. flexneri *genomes are very similar, which suggests a high level of structural and functional conservation between the two serotypes. The differences reflect different selection pressures during evolution. The ancestor of *S. flexneri *probably acquired SHI-1 and SHI-2 before SHI-O was integrated and the serotypes diverged. SHI-1 was subsequently deleted from the *S. flexneri *5b genome by recombination, but stabilized in the *S. flexneri *2a genome. These events may have contributed to the differences in pathogenicity and epidemicity between the two serotypes of *S. flexneri*.

## Background

*Shigella *species that cause bacillary dysentery or shigellosis are Gram-negative, non-sporulating, facultative anaerobes, and the disease remains a major worldwide health problem. An estimated annual infection of 160 million individuals, with 1.1 million deaths, most of them children under 5 years old in developing countries, occurs with shigellosis [1]. The poor sanitary conditions prevalent in these areas contribute to the spread of the bacteria, and the expense of antibiotics and increasing antibiotic resistance complicate treatment [2].

*Shigella *was recognized as the etiological agent of bacillary dysentery in the 1890s. It was adopted as a genus in the 1950s and sub-divided into 4 species: *S. dysenteriae, S. flexneri, S. boydii and S. sonnei *[3]. According to this taxonomy,*S. flexneri *is classified into 6 serotypes (including 13 subtypes). Most previous work on the molecular pathogenesis of *Shigella *has been carried out in *S. flexneri *serotypes 2a and 5.

In China, *S. flexneri *2a is a hyperendemic species and is responsible for approximately 50–70% of >10 million cases per year, most of them associated with epidemic and pandemic shigellosis [4]. *Shigella *pathologically invades the intestinal epithelial cells, resulting in an intense inflammatory reaction characterized by abscess formation and ulceration. All *Shigella *strains contain a large virulence plasmid that is known to encode genes necessary and sufficient for invasion [5]. The virulence plasmid from *S. flexneri *serotype 2a diverges slightly from serotype 5a [5], but the epidemicities of the two serotypes differ markedly. Chromosomal genes present in "pathogenicity islands" usually participate in the pathogenic process directly, or contribute to survival in the host environments during infection [6-10], and the expression of virulence depends on a complex regulation mechanism that involves dialog between the chromosome and the virulence plasmid [11].

We and others have previously sequenced two genomes of the most prevalent species *S. flexneri *2a (strains Sf301 and 2457T) and also completed the genomes from the other three species of *Shigella *[12-14]. All these genomes show that the extensive diversity of *Shigella *is perhaps attributable to the fact that the bacteria evolved from different strains of *E. coli *and became highly specific human pathogens through convergent evolution. A better understanding of the intra-species diversity of *Shigella *requires the availability of more whole genome sequences.

We present here the complete genome sequence of *S. flexneri *5b Sf8401 and a comparison with the *S. flexneri *2a Sf301 genome, which reveals differences in the pathogenicity islands and chromosomal rearrangements between different serotypes of this species. The comparison will facilitate understanding of the common biological processes required for infection and identify unique properties that may differentiate between them in respect of epidemicity and pathogenicity, even if the virulence plasmid is closely similar. Moreover, the comparison will provide some insight into how these pathogens have evolved.

## Results and discussion

### General features

**Figure 1:**
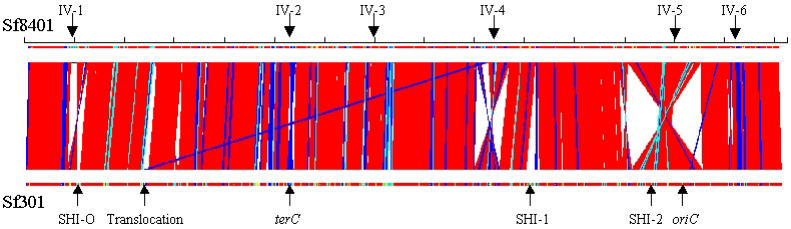
**Global comparison of Sf8401 chromosome with Sf301**. Each marker length denotes 300 kb for chromosome comparisons. Color code denotes maximal length of the paired segments: red, >10 kb; blue, 5~10 kb; cyan, 1~5 kb. The inversion is abbreviated to IV.

In common with other reported *Shigella *strains, the genome of Sf8401 contains a circular chromosome. Since the complete sequences of pWR501 and pWR100 are known [15,16], we present here only the chromosome sequence, which is 4,574,284 bp in length with an average GC content of 50.92% and encodes 97 tRNA genes (Table 1, Additional file 1). Its size is a little smaller than that of Sf301, mainly because SHI-1 is absent (see below). Sf8401 has 7 rRNA operons, with 4 copies in one replichore and 3 in the other, while Sf301 has 5 copies in one replichore and 2 in the other. These result from one intra-replichore inversion near the replication origin (Fig. 1). Comparison of Sf8401 with Sf301 reveals that more than 97% of the genome sequence is shared between the two strains. The architecture of the Sf8401 genome is similar to Sf301 but the overall colinearity is broken by 7 translocations and inversions involving DNA segments >5 kb (Fig. 1). Among the 4194 proteomes of MG1655, 3098 proteins (74%) are shared by Sf8401 and Sf301 and may be regarded as the "backbone" of *S. flexneri *(Additional file 2), while 114 are pseudogenes in both genomes. Two hundred and sixty-six of the 1096 non-backbone proteins were predicted to be metabolism-related by the Clusters of Orthologous Groups (COGs) database. This reflects the evolution of *S. flexneri *from a non-pathogenic *E. coli *ancestor to a facultative intracellular pathogen. Among the 393 Sf8401 specific proteins (compared with MG1655) only 28 are specific to all known *Shigella *genomes; most of them are bacteriophage-related proteins or hypothetical proteins (Additional file 3).

IS elements are ubiquitous in bacterial genomes and important factors in evolution [17]. The IS insertion can cause gene inactivation, activate cryptic genes or alter the expression of adjacent genes [18]. The numbers and species of IS elements in the Sf8401 genome are similar to previously-determined *Shigella *genomes. In total, the IS elements encode 485 ORFs and make up 6.37% (291.3 kb) of the chromosome, and the predominant species is IS1. A distinct difference is that Sf301 has 13 copies of iso-IS10R, while Sf8401 and other sequenced *Shigella *chromosomes have not. This might be used as a marker for epidemiological studies. Furthermore, IS elements are capable of causing various genetic rearrangements such as deletions, inversions and translocations [19,20]. Unlike the inversion reported in *Yersinia pestis *[21], *S. enterica serovar *Typhi [22] and *E. coli *K-12 strain W3110 [13,23], which are associated with rRNA homologies, inversions in Sf8401 are probably mediated by IS elements. Inversions 1 and 5 (Fig. 1) occur around SHI-O and SHI-2, which are mediated by IS629 and IS1. Besides these major inversions, there are other four inverted regions, inversions 2, 3, 4 and 6 (Fig. 1), which are probably mediated by IS4, IS*Sfl2*, IS600 and IS1 respectively.

### Diversity of SHI-O

LPS is an important virulence factor in *Shigella *[24]. Since the immune response to *Shigella *spp. is O-antigen specific, an immune response to a specific O antigen does not protect against infection with other serotypes. Therefore, the capacity to alter serotypes may be advantageous for *Shigella *spp. in the infectious process [25]. In Sf8401, the serotype conversion region, i.e. SHI-O [26], is located around 300 kb (Fig. 1). In SHI-O the 3 genes termed *gtrA*, *gtrB *and *gtrV *(a serotype-specific glycosyltransferase) are putatively involved in glucosylation reactions [27]. The mean GC content of *gtrA *and *gtrB *is 42.71%, and the GC content of *gtrV *is 32.99%. All three are lower than the whole genome GC content (50.92%). Compared with Sf301, *gtrA *and *gtrB *are highly conserved and interchangeable among serotypes, whereas *gtr *(serotype) appears to be unique to each bacteriophage [28,29], which indicates that these genes have been acquired from lysogeny caused by bacteriophages.

**Figure 2:**
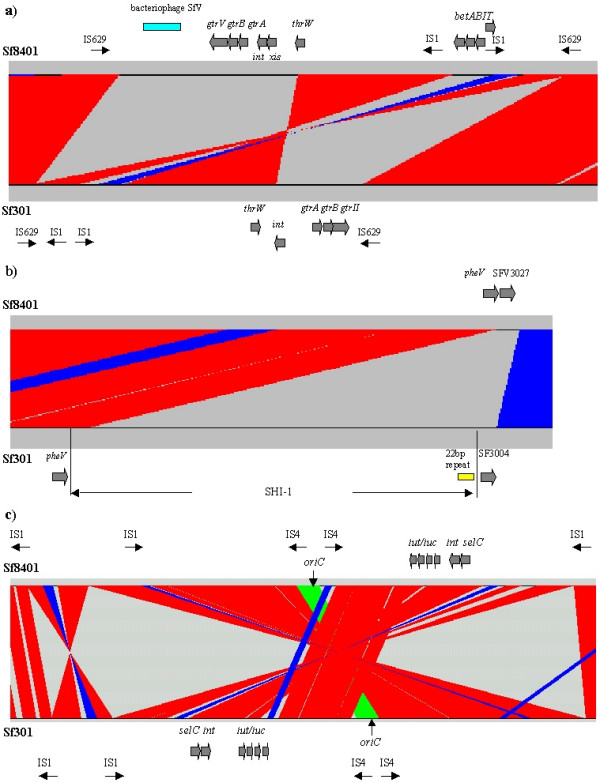
**Localized comparison of (a) SHI-O, (b) SHI-1, (c) SHI-2**. Arrows indicate predicted ORFs in both strands. The direction of each arrow predicts the direction of transcription. Color code denotes maximal length of the paired segments: red, >10 kb; blue, 5~10 kb cyan; 1~5 kb. (a) The dark green block refers to a large region of bacteriophage SfV in the Sf8401 genome. (b) The yellow block refers to the 22 bp partial repeat sequence of *pheV*. Black indicates the 12 kb sequence downstream of SHI-1 in Sf301, which does not exist in Sf8401. The asterisk denotes a pseudogene. (c) The replication origin, *ori*, is indicated by a vertical arrow in each genome. The green block refers to the region that is colinear with the Sf301 genome.

The SHI-O component differs between Sf301 and Sf8401 (Fig. 2a). Although type II antigen is encoded by an inducible bacteriophage, SfII [30], little genomic sequence of bacteriophage SfII remains in the SHI-O of 2457T and Sf301 except the key genes related to antigenic variation [13]. However, the Sf8401 SHI-O includes not only the type V antigen gene, which comes from bacteriophage SfV, but also another 15 kb segment of bacteriophage SfV [27,31].

Temperate bacteriophages of *S. flexneri *play an important role in serotype conversion. Bacteriophages SfV and SfII encode the factors involved in glucosylation of the O-antigen, and lysogenization results in conversion of serotype Y strains to serotypes 5 and 2 [27,30]. The defining point for evolution of *S. flexneri *2 and 5 from the *S. flexneri *ancestor is probably the acquisition of the precursor of the current-day O-antigen modification genes. It can be supposed that the *S. flexneri *ancestor acquired diverse O-antigen modification genes along with related bacteriophage sequences when the bacteriophages were integrated into the bacterial genomes. The genome rearrangements caused by IS elements in far-flung evolutional processes have interrupted these bacteriophage sequences in many *Shigella *genomes. However, there are ~15 kb of bacteriophage SfV sequence remnants in Sf8401, though far less bacteriophage SfII sequence has remained in Sf301. This might be because the Sf8401 genome is less dynamic than that of Sf301, or alternatively, it may suggest that Sf8401 arose later than Sf301. This hypothesis requires further investigation.

### Absence of SHI-1

*Shigella *pathogenicity island SHI-1 encodes three characterized proteins: SigA, Pic and the enterotoxin ShET1. Functional analysis shows that SigA is cytopathic for HEp-2 cells and at least partly responsible for the ability of *S. flexneri *to stimulate fluid accumulation in ligated rabbit ileal loops [6]. The Pic protein, a serine protease, is involved in mucinase activity, serum resistance and hemagglutination [9]. Furthermore, ShET1 encoded by *set1A *and *set1B *could increase fluid accumulation in the rabbit loop model.

SHI-1 is located directly downstream of the *pheV *tRNA gene and includes an imperfect repeat of the 3'-end 22 bp of the *pheV *gene at the right boundary in Sf301 (Fig. 2b). Studies of SHI-1 distribution (*she *PAI) show that intact SHI-1 is present in all tested serotype 2a strains of *S. flexneri *but absent from some *S. flexneri *serotype strains such as 1a, 1b, 3b, 4 and 5 [32]. It is therefore not surprising that SHI-1 is wholly absent from Sf8401. However, the homolog of SF3004, a hypothetical protein located downstream of SHI-1 in Sf301, is situated next to the *pheV *gene in Sf8401 (SFV3027). Further investigation shows that the homolog of SF3004 is only present adjacent to SHI-1 in the Sf301 and 2457T genomes and the *S. flexneri *2a *she *pathogenicity island [6]. So the presence of SFV3027 suggests that Sf8401 might have contained SHI-1 during its evolutionary history but lost it for unknown reasons. Sakellaris et al. [33] demonstrated that the spontaneous and precise excision of SHI-1 can occur via recombination between a 22 bp sequence at the 3' terminus of *pheV *and an imperfect direct repeat at the *pheV*-distal boundary of the SHI-1. Thus, we have probably witnessed the case in Sf8401. Undoubtedly, the mechanism by which SHI-1 is stabilized in *S. flexneri *2a strains will be an interesting focus for further studies.

### Inversion of SHI-2

SHI-2 encodes the synthesis and transport of aerobactin, a hydroxamate siderophore associated with increased virulence in enteric bacteria [10,34], and is located downstream of the *selC *tRNA gene in Sf8401. The conservation of the component, organization and integration site of SHI-2 in Sf301 and Sf8401 implies that it was acquired by the *S. flexneri *ancestor before the serotypes diverged. However, the *iut*/*iuc *operon is located on the leading strand and on the counter-clockwise site of the replication origin in Sf301, but on the lagging strand and on the clockwise site in Sf8401 (Fig. 2c). Our previous genome studies revealed that SHI-2 in *S. sonnei *Ss046 was unlinked with the *selC *gene by an inversion, and SHI-3 of *S. boydii *Sb227 that carries a similar *iut*/*iuc *operon is linked with the *pheU *tRNA locus [14]. So in view of this information, the observation that the *iut*/*iuc *operon can inserted into a variety of different loci suggests that it is highly mobile and may be acquired by additional human or animal pathogens [10].

In Sf8401, SHI-2 is involved in inversion 5 that spans *oriC *(Fig. 1). All sequenced *Shigella *chromosomes have inversions at *oriC *and *terC*, which is suggested to be a common evolutionary feature of bacterial genomes [35]. In contrast to Sf301, inversion 5 is found from 3597 to 4100 kb in Sf8401, which appears to be mediated by the boundary IS1 copies in Sf8401 (Fig. 2c). However, the ~22 kb center region of inversion 5 that covers *oriC *retains colinearity with that of Sf301 (Fig. 2c; the block colored green). Since this region is sandwiched between two copies of IS4, it implies that two or more inversions have occurred: an inversion was followed by a re-inversion to restore colinearity.

Inversions can produce an "X" shape, which changes the positions of these sequences from their natural locations [35], and the distance from the *iut/iuc *operon to *oriC *in Sf8401 is different from that in Sf301 due to inversion 5 and the internal re-inversion mentioned above. Owing to bidirectional replication, there are extra copies of genes close to *oriC*, resulting in increased gene expression [36], and since dosage differences may cause the strengths of promoters to be evolutionarily optimized for their specific positions, cells in which genes are a different distance from *oriC *are at a selective disadvantage [37]. Hence, whether the change in position of the *iut/iuc *operon in Sf8401 has any influence on expression needs to be determined.

### Differences among genes correlated with metabolism

The *E. coli bet *gene cluster (*betABIT*) contributes to the pathway for glycine-betaine biosynthesis from choline [38] and is located close to SHI-O in Sf8401 but absent from Sf301 and 2457T. Among eubacteria-compatible solutes, the most widespread is glycine-betaine, and this is the only osmoprotectant synthesized by *E. coli *[39,40]. The choline-glycine-betaine pathway confers a high level of osmotic tolerance on *E. coli *[38]. Whether this system offers an advantage to *S. flexneri *5b for environmental survival over *S. flexneri *2a or plays a role during infection requires further investigation. However, the inversion around the SHI-O may contribute to the deletion of the *bet *operon from *S. flexneri *2a (Fig. 2a).

There are some differences in metabolic and physiological pathways between Sf8401 and Sf301. In both strains, some key metabolic pathways were inactivated by the creation of independent pseudogenes. For instance, the loss of ability to utilize D-sorbitol is due to the inactive states of *srlE *and *srlA *in Sf8401 and Sf301, respectively. It seems that *Shigella *strains have a general tendency to lose some pathways and functions, and this tendency has given rise to convergent evolution [41]. The microorganisms need to adapt to new niches by adopting a strictly pathogenic life-style. There must be some genes that provide little overall selective benefit in a new situation. These genes will be eliminated through mutational bias favoring deletions for the lack of selective force to maintain them [42]. Such functions, no longer active in one serotype but expressed in another, may lead to a better understanding of the diversity of the two serotypes and the evolution of *S. flexneri*.

## Conclusion

As more bacterial genomes have been sequenced during recent years, the study of comparative genomics has progressed rapidly. Although five *Shigella *genomes have been reported, this is the first time that intra-species diversity has been characterized by comparing the genomes of two different serotypes of the same *Shigella *species. The comparison between Sf8401 and Sf301 has provided abundant biological and medical information.

The overall genomic organization, gene order and predicted proteomes of the two genomes are very similar, which suggests a high level of structural and functional conservation between the serotypes. Nevertheless, the colinearity of genome structure between these two serotypes was disrupted by several inversions, and along with the differences found in the known PAIs, these may contribute to differences in epidemicity and virulence between *S. flexneri *5b and *S. flexneri *2a.

*S. flexneri *5b and *S. flexneri *2a have experienced different selection pressures and evolutionary processes. These events (such as inversion, translocation, deletion and acquisition) have led to the diversity of SHI-O, the absence of SHI-1, the shift of SHI-2, and other differences between these two genomes. They have extensively reshaped the genome, presumably virulence to be more fully expressed. It can be supposed that the *S. flexneri *ancestor had acquired SHI-1 and SHI-2 before divergence, and diverged into different serotypes after the different SHI-Os were integrated. Subsequently, *S. flexneri *5b deleted SHI-1 via recombination and changed the structure of SHI-2 by inversions, but *S. flexneri *2a stabilized SHI-1 in its genome. Mechanisms not yet identified have helped to shape the differences between serotypes and led to the derivation of different serotypes from the same parental *S. flexneri *form. Characterizing the divergence between serotypes at the genetic level helps us to understand the evolution of *S. flexneri*. The interplay between organizing features of the chromosome, such as the pathogenicity and the elements inducing sequence variation and chromosomal rearrangements, may provide an explanation of why different genomes show such different levels of organization and how this relates to their evolutionary history and ecology.

In conclusion, by comparing the genomes of Sf8401 and Sf301, a large amount of data has been obtained. It is important in biological and medical research to compare genomes causing similar types of diseases in the same subspecies but different serotypes. The identification of shared traits is important for pathogenicity and for the study of its conservation, transfer, epidemiology, virulence and evolution. Although it is not clear at present whether these similarities and differences are common among *S. flexneri*, there is a possibility that such events lead to differences in virulence and pathogenicity. Future studies should identify which of the differences in these genomes accounts for the phenotypic differences.

## Methods

### *Shigella flexneri *5b strain

*Shigella flexneri *5b, strain 8401, was isolated and sequenced from epidemic in China, kindly provided by the National Institute for Communicable Disease Control and Prevention, Chinese Centre for Disease Control and Prevention.

### Shotgun sequencing and analysis

The whole genome sequence shotgun libraries for Sf8401 were established as described previously [12,14], and ABI3730 automated sequencers were used for sequence collection. 48,000 clones were sequenced from both ends, giving rise to 8 times coverage of the genome. Sequences were assembled initially using the phred/phrap program with the Q20 criteria [43] when the sequence coverage was ~4-fold over the estimated size of the genome. The Consed program was used for sequence finishing [43]. Gaps among contigs were closed either by primer walking on selected clones, which were identified by analysis on the forward and the reversed links between contigs using a perl/Tk script, or by sequencing the DNA amplicons generated by polymerase chain reaction (PCR). Glimmer 2.0, a program that searches for protein coding regions, was used to identify those ORFs possessing more than 30 consecutive codons [44]. Overlapping and closely clustered ORFs were manually inspected. Predicted polypeptide sequences were used to search the non-redundant protein database with BLASTP, and the clusters of orthologous groups of proteins (COGs) database was used to identify families to which predicted proteins are related [45]. Those cases in which a stop condon or deletion has resulted in an encoded protein that is less than 80% of the length of its counterpart in K-12 genome and those cases in which a frameshift of insertion has altered more than 20% of the amino acid sequence were classed as pseudogenes. Mobile elements and repetitive sequences were identified using the IS FINDER database [46]. GenomeComp was used for genomic comparison with default parameters [47]. The comparison figures used in Figure 1 and 2 were exported from GenomeComp with a 1500 bp filter setting along with the scale setting of 2000 for chromosomes. The KEGG database was used for the metabolic pathways analysis [48].

### Data accessibility

Complete genome sequence of Sf8401 has been deposited in the Genbank. The accession number for chromosome is: CP000266.

## Authors' contributions

HN, carried out the molecular genetic studies, participated in the sequence alignment and drafted the manuscript. JY, FY, LC performed the statistical analysis and comparative genomic analysis. JY, XZ, JP participated in manuscript preparation. FY, JW, ZX participated in the sequence alignment. LLS, JD, YX, XX, and SC contributed to sample preparation for shotgun sequencing. ZY, YS contributed for design of the manuscript. QJ participated in the design and helped to draft the manuscript. All authors have read and approved the manuscript.

## Supplementary Material

Additional file 1 **Circular genome map of the Sf8401 genome**. The outer scale is marked every 200 kb. Circles range from 1 (outer circle) to 9 (inner circle). Circles 1 and 2, ORFs encoded by leading and lagging strands respectively, with color code for functions: salmon, translation, ribosomal structure and biogenesis; light blue, transcription; cyan, DNA replication, recombination and repair; turquoise, cell division; deep pink, post-translational modification, protein turnover and chaperones; olive drab, cell envelope biogenesis; purple, cell motility and secretion; forest green, inorganic ion transport and metabolism; magenta, signal transduction; red, energy production; sienna, carbohydrate transport and metabolism; yellow, amino acid transport; orange, nucleotide transport and metabolism; gold, co-enzyme transport and metabolism; dark blue, lipid metabolism; blue, secondary metabolites, transport and catabolism; gray, general function prediction only; black, function unclassified or unknown. Circle 3, distribution of pseudogenes. Circles 4 and 5, distributions of IS1/IS1N and other IS-species respectively. Circles 6 and 7, G+C content and GC skew (G-C/G+C) respectively with a window size of 10 kb. Circles 8 and 9, distributions of tRNA genes and *rrn *operons respectively. The replication origin and terminus are indicated. Click here for file

Additional file 2 **Orthologs of the MG1655 proteomes in Sf8401 and Sf301**. 4194 proteins of MG1655 are selected to study their orthologs in Sf8401 and Sf301. Click here for file

Additional file 3 **Orthologs of Sf8401 specific proteins (relative to MG1655) in other *Shigella *genomes**. This gives the full list of 393 Sf8401-specific proteins relative to MG1655 and their orthologs in other *Shigella *genomes. Click here for file

## Figures and Tables

**Table 1:** General features of the *Shigella *genomes compared with the genome of *E. coli *K12 MG1655

**Chromosome**	MG1655*	**Sd197† **	**Sf301† **	**Sf8401**	**Sb227† **	**Ss046† **
Total length (bp)	4,639,675	4,369,232	4,607,203	4,574,284	4,519,823	4,825,265
No. of total ORFs	4,254	4,557	4,434	4,316	4,353	4,434
No. of pseudogenes	12	285	254	198	217	210
Percentage of CDS (%)	87.3	77.2	80.4	80.8	80.5	80.5
G+C content (%)	50.79	51.25	50.89	50.92	51.21	51.01
No. of 16S/23S/5S	7/7/8	7/7/8	7/7/8	7/7/8	7/7/8	7/7/8
No. transfer RNA	86	85	97	97	91	97
IS elements (percentage)	44 (1%)	623 (12%)	314 (7%)	278 (6.3%)	403 (9%)	394 (8%)

* Data are obtained from a recently updated version of U00096.

† Data are obtained from ref.[14].
