# Supplementary figures and images for: Complete genome sequence of Shigella flexneri 5b and comparison with Shigella flexneri 2a
Source: BMC Genomics. 2006 Jul 6;7:173. doi: 10.1186/1471-2164-7-173 (PMC1550401; doi:10.1186/1471-2164-7-173)

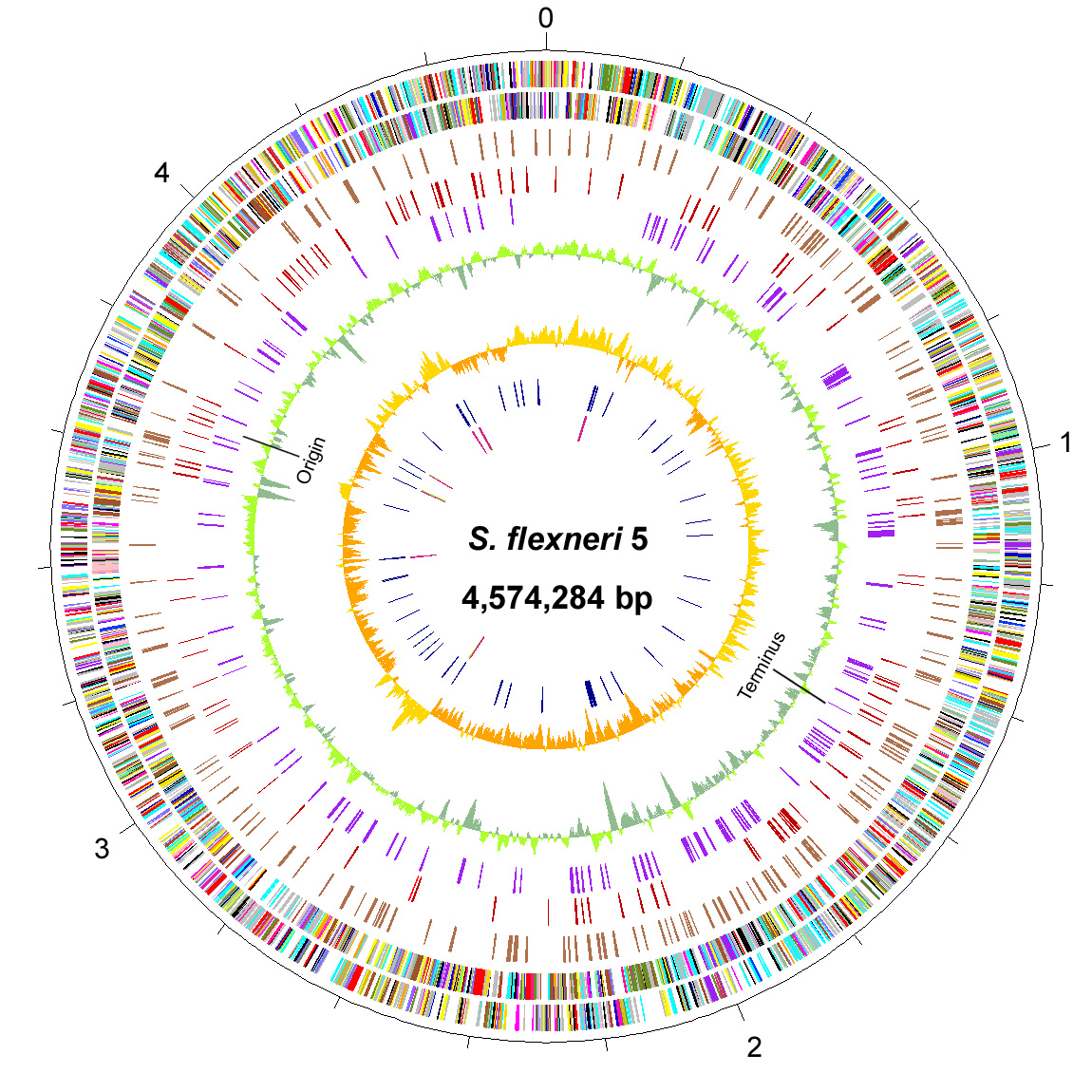

Supplement: Additional file 1 — Circular genome map of the Sf8401 genome. The outer scale is marked every 200 kb. Circles range from 1 (outer circle) to 9 (inner circle). Circles 1 and 2, ORFs encoded by leading and lagging strands respectively, with color code for functions: salmon, translation, ribosomal structure and biogenesis; light blue, transcription; cyan, DNA replication, recombination and repair; turquoise, cell division; deep pink, post-translational modification, protein turnover and chaperones; olive drab, cell envelope biogenesis; purple, cell motility and secretion; forest green, inorganic ion transport and metabolism; magenta, signal transduction; red, energy production; sienna, carbohydrate transport and metabolism; yellow, amino acid transport; orange, nucleotide transport and metabolism; gold, co-enzyme transport and metabolism; dark blue, lipid metabolism; blue, secondary metabolites, transport and catabolism; gray, general function prediction only; black, function unclassified or unknown. Circle 3, distribution of pseudogenes. Circles 4 and 5, distributions of IS1/IS1N and other IS-species respectively. Circles 6 and 7, G+C content and GC skew (G-C/G+C) respectively with a window size of 10 kb. Circles 8 and 9, distributions of tRNA genes and rrn operons respectively. The replication origin and terminus are indicated. [file 1471-2164-7-173-S1.jpeg]
